# Impact of E1 and Cre on Adenovirus Vector Amplification: Developing MDCK CAV-2-E1 and E1-Cre Transcomplementing Cell Lines

**DOI:** 10.1371/journal.pone.0060342

**Published:** 2013-04-02

**Authors:** Paulo Fernandes, Virgínia M. Santiago, Ana F. Rodrigues, Hélio Tomás, Eric J. Kremer, Paula M. Alves, Ana S. Coroadinha

**Affiliations:** 1 iBET, Instituto de Biologia Experimental e Tecnológica, Oeiras, Portugal; 2 Instituto de Tecnologia Química e Biológica, Universidade Nova de Lisboa, Oeiras, Portugal; 3 Institut de Génétique Moléculaire de Montpellier, CNRS – Universities of Montpellier I and II, Montpellier, France; Justus-Liebig-University Giessen, Germany

## Abstract

Adenovirus vectors have been extensively studied through the manipulation of viral genome. However, little attention is being paid to their producer cell-lines; cells are selected according to virus yields, neglecting the expression profile of transcomplementing gene products underlying cell performance. This work evaluates the impact of E1 (E1A and E1B) and Cre recombinase levels in the production of E1-deleted and helper-dependent canine adenovirus type 2 (CAV-2) vectors using MDCK cells. E1A and E1B gene expression and Cre activity were evaluated in different cell clones and compared with the corresponding cell productivity and susceptibility to oxidative stress injury. CAV-2 production was proportional to E1A expression (the highest levels of E1A corresponding to productivities of 3000–5000 I.P./cell), while E1B prolonged host cell viability after infection, conferring protection against apoptosis. Cre recombinase counteracted E1B anti-apoptotic properties, however viral production was maintained under high levels of Cre. Yet, Cre recombinase side effects can be reduced using cell lines with lower Cre-activities, without compromising the excision efficiency of helper vector packaging signal. These results highlight the influence of transcomplementing gene products on CAV-2 producer cell line performance, and the ability to express high levels of E1A and E1B as an important feature for cell line establishment and high adenovirus titers.

## Introduction

Adenovirus vectors (AdV) are efficient gene transfer vectors due to the ability to efficiently infect a wide variety of quiescent and proliferating cell types leading to high-level gene expression [1]. These vectors have been extensively studied for gene therapy applications, where the AdV genome has been progressively modified from the wild-type to improve its safety and efficacy in therapeutic applications. However, little attention has been paid to the producer cell lines. So far, the main efforts during cell line development are focused in avoiding the generation of replicative-competent adenovirus (RCA) by sophisticated designs of the transforming plasmid; afterwards, cell lines that allow high-titers of AdV are selected (reviewed in [2]). Thus, the expression profile of transcomplementing genes behind cell lines performance has been neglected.

Most replication-defective adenovirus vectors require, for manufacturing and replication, a cell line that expresses the adenoviral E1 functions in *trans*. E1 region codes for two subunits – E1A and E1B – important to direct cellular and viral gene expression to enable a productive virus cycle [3]. E1A function is crucial for viral DNA replication; it transactivates other early units and deregulates various cell cycle controls. E1A gene is composed by two exons and several E1A polypeptides are produced following alternative splicing of a primary RNA transcript. The most abundant E1A proteins are derived from differentially spliced 12S and 13S mRNA that give rise to two proteins of variable size, depending on the adenovirus serotype and species, and act as major regulators of early viral transcription activating other early promoter regions [3], [4]. Adenovirus E1A gene expression products stimulate infected cells to enter S phase of the cell cycle and provide an intracellular environment for viral replication. The E1A also contributes to the transforming ability attributed to the virus, but requires the expression of E1B [5], [6]. E1B gene encodes two major proteins generally designated according to adenovirus terminology by E1B 19 kDa and E1B 55 kDa, whose main functions are inhibition of apoptosis, protection of viral and cellular DNA from degradation during infection and further intracellular environment modification, to make the cell more hospitable to viral protein production and viral DNA replication [7]–[10].

From vectors with the deletion of the E1 region to helper-dependent AdV with the deletion of viral genes, an enhanced capacity for a gene therapeutic insertion from ∼7 kb to ∼36 kb has been achieved [11]. Helper-dependent vectors (HDV) harbors the inverted terminal repeats (ITRs), where replication starts, the packaging signal (Ψ), and an expression cassette. For that reason, their production requires trans-acting elements provided by an helper vector (usually a ΔE1 vector) after co-infecting cells. To prevent helper vector (HV) packaging, during HDV production, the Ψ sequence can be removed by flanking the Ψ with recombinase recognition sequences that are recognized by the recombinase stably expressed in the transcomplementing cell line. Therefore, the transcomplementing cell line must also express a recombinase, such as Cre recombinase [12]–[14].

In this work, we studied the impact of E1 expression in the development of MDCK-E1 and MDCK-E1-Cre cell lines for E1-deleted and HD canine adenovirus type 2 (CAV-2) vectors. Although human AdV is generally the preferred prototype vector backbone, memory immunity (humoral and cellular) may limit the efficiency in humans [15]–[21]. One alternative to circumvent this drawback, while keeping other AdV advantages, is the use of non-human adenovirus. CAV-2 vectors, showing longer transgene expression due to the absence of neutralizing CAV-2 antibodies in the serum of healthy human individuals [20]–[23], preferentially transduce neurons and have a remarkable capacity of axonal transport, making them promising tools for the treatment of neurodegenerative diseases [22], [24], [25]. To investigate the production of CAV-2 vectors under different levels of E1 (E1A and E1B) different cell-clones were analyzed. This work confirms similar function of adenovirus E1 proteins, predicted by conserved sequences [26]. We show that viral replication was influenced by the levels of E1A and E1B gene products. Higher levels of Cre recombinase, although counteracting E1B anti-apoptotic properties, had no effect on viral yields. Mechanisms underlying virus production and its relationship with E1A, E1B and Cre are discussed in the context of cell line development.

## Materials and Methods

### Plasmids

For the transfection of MDCK with E1 genes, pCI-neo plasmid backbone containing CAV-2 E1A gene under the CMV promoter control and CAV-2 E1B gene under the control of its own promoter was used and named pCI-NeoK9 [16], [20]. E1 region corresponds to the sequence 354–3609 bp available in GenBank accession no. J04368 [20]. Additionally, pCI-NeoK9 confers neomycin resistance allowing the selection of cells after transfection. Cre recombinase expression was obtained with pZeoCre plasmid [23] conferring Zeocin resistance and in which Cre was cloned under the control of SV40 promoter.

### Cell Lines and Culture Media

MDCK cells (ECCAC, Nr 84121903), MDCK-E1 clones and MDCK-E1-Cre subclones were grown in Dulbecco’s Modified Eagle’s Medium (DMEM) (Gibco, Paisley, UK) supplemented with 10% (v/v) FBS (Gibco). Dog Kidney cells expressing E1 from CAV-2 (DKZeo) and expressing E1 and Cre (DKCre) [20], [23] were used as control cells and maintained in serum-supplemented DMEM (Gibco). All cells were maintained at 37°C in a humidified atmosphere air containing 5% CO_2_.

Cell concentration and viability was determined by the trypan blue exclusion method using a 0.1% (v/v) solution prepared in PBS and counting cells in a Fuchs-Rosenthal haemacytometer (Brand, Wertheim, Germany).

### Vectors

CAVGFP, JBΔ5 and HD CAVGFP are vectors derived from CAV-2 strain Toronto A 26/61, GenBank U77082. CAVGFP [20] and HD CAVGFP [25] are E1-deleted (ΔE1) and HD vectors, respectively, and contain an eGFP expression cassette. JBΔ5 is a helper vector containing loxP flanking the packaging domains and a RSV-*lacZ* expression cassette [25]. For CAVGFP and JBΔ5 viral stocks preparation, 150 cm^2^ T-flasks with DKZeo cells at a confluency of 80–90% were infected with a MOI of 5 infectious particles (I.P.) per cell with medium exchange at the time of infection. 40 hours post infection (hpi) cells were collected and lysed with 0.1% (v/v) Triton 100 (Sigma-Aldrich, Steinhein, Germany) in Tris-HCl 10 mM, pH 8. The lysate was clarified by centrifugation at 3000 *g* during 10 min at 4°C and purified by CsCl gradients as described previously [20]. The purified vectors were stored in phosphate buffered saline (PBS) with 10% (v/v) glycerol in aliquots at −85°C. HD CAVGFP viral stock used in this work was prepared from a purified HD CAVGFP obtained as described previously [25]. Then, two amplification rounds were performed by infecting DKCre cells, using an MOI ratio between HD CAVGFP and JBΔ5 of 5 to 1. Viral vectors from the second amplification round were purified as mentioned above.

### Establishment of MDCK-E1 and MDCK-E1-Cre Clones

MDCK cells were transfected in suspension using 10 µg of polyethylenimine (PEI) (Polysciences, Eppelheim, Germany) *per* 1×10^6^ cells, with the plasmid pCI-NeoK9 (4 µg). Selection was performed in medium containing 500 µg/mL of Geneticin (G418) (Invivogen, Toulouse, France). This cell population was cloned by limiting dilution with 50% (v/v) conditioned medium, 20% FBS (v/v) and 250 µg/mL of G418 (Invivogen). 121 clones were isolated. MDCK-E1-Cre subclones were generated using a similar protocol; after transfecting MDCK-E1#106 cell clone with pZeoCre, the cells were selected using 500 µg/mL of Zeocin (Invivogen) followed by cloning by limiting dilution.

### E1 Gene Expression Analysis

**Figure 1:**
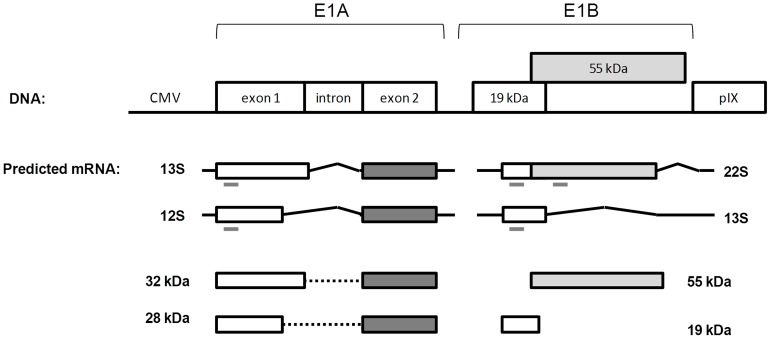
Schematic representation of the DNA E1 region used to express in MDCK-E1 transcomplementing cells and the predicted major mRNA and protein derived from E1A and E1B genes. Predicted mRNAs and proteins were inferred by sequence homology [26] with human adenovirus. E1A 32 kDa and 28 kDa are translated from 13S and 12S mRNAs from E1A respectively. E1B 55 kDa is translated from 22S mRNA and E1B 19 kDa protein can be translated from the 22S or 13S mRNAs from E1B. Grey dashes underlying the mRNA products represent the generated PCR amplicon in the expression analysis of E1A and E1B genes. One set of primers was designed to assess E1A expression since it is expected that most generated splicing transcripts will contain the initial 5′ region of exon 1. Two sets of primers were design for E1B to confirm the formation of mRNA transcripts containing the 55 KDa open reading frames.

RNA samples were extracted from 1×10^6^ cells of MDCK, DKZeo and MDCK-E1 clones using RNeasy mini kit (QIAGEN, Germantown, MD, USA). cDNA synthesis from extracted RNA was performed using “Transcriptor first strand cDNA synthesis kit” (Roche Diagnostics, Mannheim, Germany). cDNA synthesis mix was prepared to the manufacturer’s indicated end-concentrations. Amplification of cDNA was performed using Fast Start PCR Master kit (Roche Diagnostics). Detection of E1A and E1B (for E1B 19 kDa and E1B 55 kDa) genes was performed using the following primers: for E1A gene sense primer sequence 5′-CCGCGCAATCTCCATGATTA-3′ and antisense primer sequence 5′-AGTGCTCGCACTCGAATCAG-3′; for E1B 19 kDa sense primer sequence 5′-TACTTTGTCGCCTGGATTTT-3′ and antisense primer sequence 5′-CTGTCTACCTCTATTTTCCAGC-3′ and for E1B 55 kDa sense primer sequence 5′-CACACTTTAGAAATGCCCAG-3′ and antisense primer sequence 5′-CCGTAACCCTAATCTTAGAA-3′. The primers used for E1A gene were designed to consider both mRNA transcripts originating E1A 12S and E1A 13S RNAs, since they amplify in exon 1 containing CR1 and CR2 regions present in both transcripts (**Figure 1**). E1B codes for two unrelated proteins, the 19 kDa and 55 kDa proteins, with partially overlapping reading frames. Thus two different pairs of primers against non-overlapping regions accounting for the two different mRNA transcripts were designed (**Figure 1**). GAPDH (Glyceraldehyde 3-phosphate dehydrogenase) was selected as the endogenous control gene and was amplified using sense primer sequence 5′-AACATCATCCCTGCTTCCAC-3′ and antisense primer sequence 5′-GACCACCTGTTCCTCAGTGT-3′.

For LightCycler real time PCR a master mix with the following reaction components was prepared to the indicated end-concentrations: 2 µL LightCycler master (Fast start DNA master SYBR Green I; Roche Diagnostics), 2.4 µL MgCl_2_ (3 mM), 2 µL forward and reverse primer (0.3 µM), and 9.6 µL PCR grade water. LightCycler master mix was distributed into the LightCycler capillaries (18 µL each) and either 2 µL of pCI-neoE1K9, pGAPDH (standard curves for E1 and GAPDH gene) or cDNA was added. The following LightCycler run protocol was used: denaturation program (95°C, 10 minutes); amplification and quantification program repeated 40 times (95°C for 10 seconds; 60°C (E1A gene) and 55°C (E1B 19 kDa, E1B 55 kDa) for 10 seconds; 72°C for 9 seconds); melting curve program (95°C 0 seconds; 65°C for 15 seconds; 95°C 0 seconds); and finally a cooling step to 40°C. The results obtained were analyzed with LightCycler software 4.1 (Roche Diagnostics).

### Cre Activity

Relative Cre activity was assessed by the luciferase activity generated by the adenovirus vector AdMA19 after infecting cells [27]. AdMA19 contains the luciferase cDNA under control of the human cytomegalovirus promoter but separated from it by an extraneous spacer sequence composed by a series of initiation and stop codons in several reading frames. This translational “start/stop sign”, flanked by loxP, disrupts translation. If the cell clone expresses Cre, the stop sign is floxed out and translation proceeds leading to luciferase expression. Briefly, cells were seeded in 24 well-plates in quadruplicates and infected the day after with 20 I.P./cell of AdM19 with medium exchange. Twenty-four hpi, cells were counted and lysed to release luciferase, both in duplicates. The resulting supernatant was collected and the light units were quantified with a Modulus Luminometer from Turner Biosystems (Sunnyvale, CA, USA) after adding luciferin and normalized to the cell concentration.

### Oxidative Stress Induction and Cell Viability Assessment

The susceptibility to oxidative stress injury was assessed by analyzing *tert*-butyl hydroperoxide induced death rates. Briefly, cells were inoculated at 0.8×10^4^ cells/cm^2^ in 24 well plates. At the 2^nd^ day of culture, when cells reached approximately 90% of confluence, *tert*-butyl hydroperoxide (Sigma) was added in increasing concentrations ranging from 1.3 to 608.4 µM. Cell viability was quantified 18 h later by negative propidium iodide (Sigma) staining and IC_50_ values calculated by non-linear fitting with a 95% confidence interval using Graphpad Prism (Graphpad Software, Inc., La Jolla, CA, USA) and same approach as the one previously described [28].

### CAVGFP, JBΔ5 and HD CAVGFP Amplification by MDCK Cells

Cell clones were screened for CAVGFP vector amplification in tissue-culture Petri dishes with 10 cm diameter (BD Biosciences, USA). MDCK-E1 clones were seeded at 1.5×10^4^ cells/cm^2^, infected the day after with a MOI of 5 I.P. and incubated for further 40 hours. CAVGFP vectors were collected using triton (Sigma-Aldrich) 0.1% (v/v) in Tris-HCl, clarified at 3000 *g* for 10 min at 4°C and stored at −85°C until further analysis. To evaluate the production decrease of helper JBΔ5 under the presence of Cre, MDCK-E1-Cre subclones, DKCre cells and corresponding parental cells were infected with 5 I.P./cell of JBΔ5 using the same protocol described above. The production assays for HD CAVGFP was performed accordingly, by infecting cells with 5 I.P./cell of HD CAVGFP and 1 I.P./cell of JBΔ5.

### Infectious Vectors Titration

Quantification of infectious CAVGFP and HD CAVGFP was performed by monitoring the expression of GFP. MDCK cells were seeded in 24-well plates at 2×10^4^ cells/cm^2^ and infected the day after using serial dilutions of viral suspensions in fresh DMEM (Gibco). Infected cells were harvested 24 hpi and the percentage of GFP-positive cells determined by flow cytometry (CyFlow Space, Partec, Germany). Two replicates were performed for each dilution. CAVGFP titer was determined by multiplying the number of cells at infection with the percentage of GFP expressing cells and the respective dilution factor.

Titration of infectious JBΔ5 vectors was based in lacZ gene expression and β-galactosidase activity by adapting a previously described protocol [29]. Briefly, DKZeo cells were seeded at 1×10^5^ cells/cm^2^ in 96-well plates. After 24 h, cells were infected with serial dilutions of viral suspension. The day after, infected cells were fixed and stained using a solution with X-gal (5-bromo-4-chloro-3-indolyl-b-D-galactopyranoside) (Stratagene, La Jolla, CA, USA). Four replicates were performed for each dilution. JBΔ5 titer was determined by counting the stained blue cells using an inverted phase contrast microscope, multiplied by the dilution factor.

Productivities were shown as amplification ratio, corresponding to the ratio between I.P. harvested and those used at infection (I.P. Out/I.P. In), or as cell specific viral titers, corresponding to total I.P. normalized to cell concentration at virus harvesting time.

### Statistical Analysis

Statistics were performed using Microsoft Office Excel and Graphpad Prism software. Data are presented as mean ± standard-deviation. Statistical significance was determined by single factor Anova analysis with P value set at <0.05.

## Results

### E1 Gene Expression and ΔE1 CAV-2 Amplification

MDCK cells were stably transfected with the plasmid encoding E1 genes from CAV-2 (**Figure 1**) and clones with different expression levels were obtained. To evaluate the ability of the clones to produce ΔE1 CAV-2 vectors, a production assay using CAVGFP was performed. Nine of the cell clones efficiently propagated CAVGFP with productivities above 300 I.P./cell.

**Figure 2:**
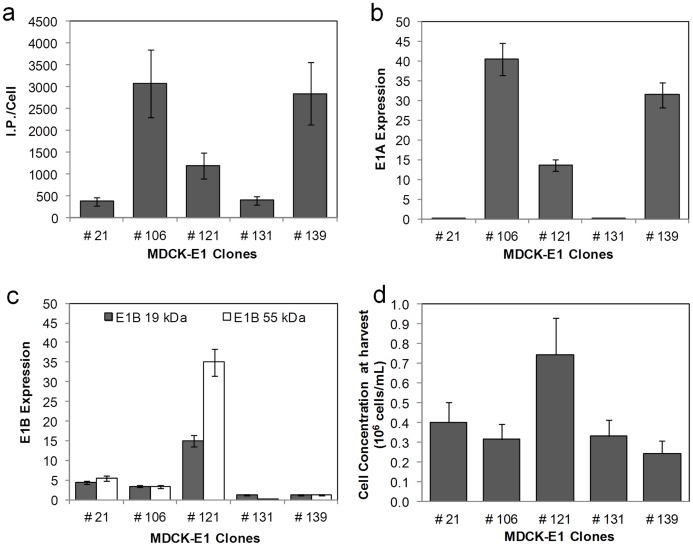
Cell specific productivity and corresponding E1 gene expression of MDCK-E1 cell clones. **a.** Cell specific infectious viral titer produced in MDCK-E1 clones using DMEM 10% (v/v) FBS and 1% (v/v) NEAA. Error bars represent a 25% inter-assay variability error. **b and c.** Levels of mRNA E1A (**b**) and E1B expression (**c**) obtained for the different MDCK-E1 clones. The gene expression was determined by Real Time reverse transcriptase PCR and normalized to the housekeeping gene (GAPDH). Error bars represent a 10% variability error associated with the method. **d.** Cell concentration obtained for the different MDCK-E1 clones at virus harvest time. Cells were infected at the same cell concentration. Error bars represent the standard deviation of three independent experiments (n = 3).

To assess the effect of E1 expression on virus replication, five MDCK-E1 clones showing different levels of virus productivities were analyzed (**Table 1**): MDCK-E1#106 and MDCK-E1#121 had high amplification yields of 469 and 497 (I.P. Out/I.P. In) respectively, MDCK-E1#139 had an intermediate amplification yield of 349, and finally MDCK-E1#21 and MDCK-E1#131 had amplification yields below 100. Although MDCK-E1#121 presented higher amplification yields than clones #106 and #139, attained lower cell specific virus productivity (I.P./Cell) (**Figure 2a**). This can be justified by the significant higher cell density at the virus harvesting time of MDCK-E1#121 (**Figure 2d**).

**Table 1:** Virus amplification in MDCK-E1 clones and DKZeo cells.

Cells	Amplification ratio
MDCK-E1#21	94±23
MDCK-E1#106	469±117
MDCK-E1#121	497±124
MDCK-E1#131	92±23
MDCK-E1#139	349±87
DKZeo	984±246

The error corresponds to 25% inter-assays variability.

E1 region expression was investigated by determining the mRNA levels of E1A and E1B transcripts. No relation between E1A and E1B expression was observed. Lower expression of E1B versus E1A was obtained because the former is driven by its own weaker promoter. The highest E1A gene expression was obtained for clone MDCK-E1#106, followed by clone #139 and #121 (**Figure 2b**). MDCK-E1#21 and #31 had the lowest levels of E1A expression. Concerning E1B expression, MDCK-E1#121 had levels on the average 3- and 7-fold more than the other clones for E1B 19 kDa and E1B 55 kDa, respectively (**Figure 2c**).

A correlation between E1A mRNA expression (**Figure 2b**) and cell specific viral production (**Figure 2a**) can be observed, suggesting that higher E1A expression favors vector production. On the other hand MDCK-E1#121 presented a 2-fold increase in viable cells concentration at virus harvesting time (**Figure 2d**), which may indicate cell survival advantages. E1B contribution for vector production may thus be indirect by preventing loss of cell viability.

**Figure 3:**
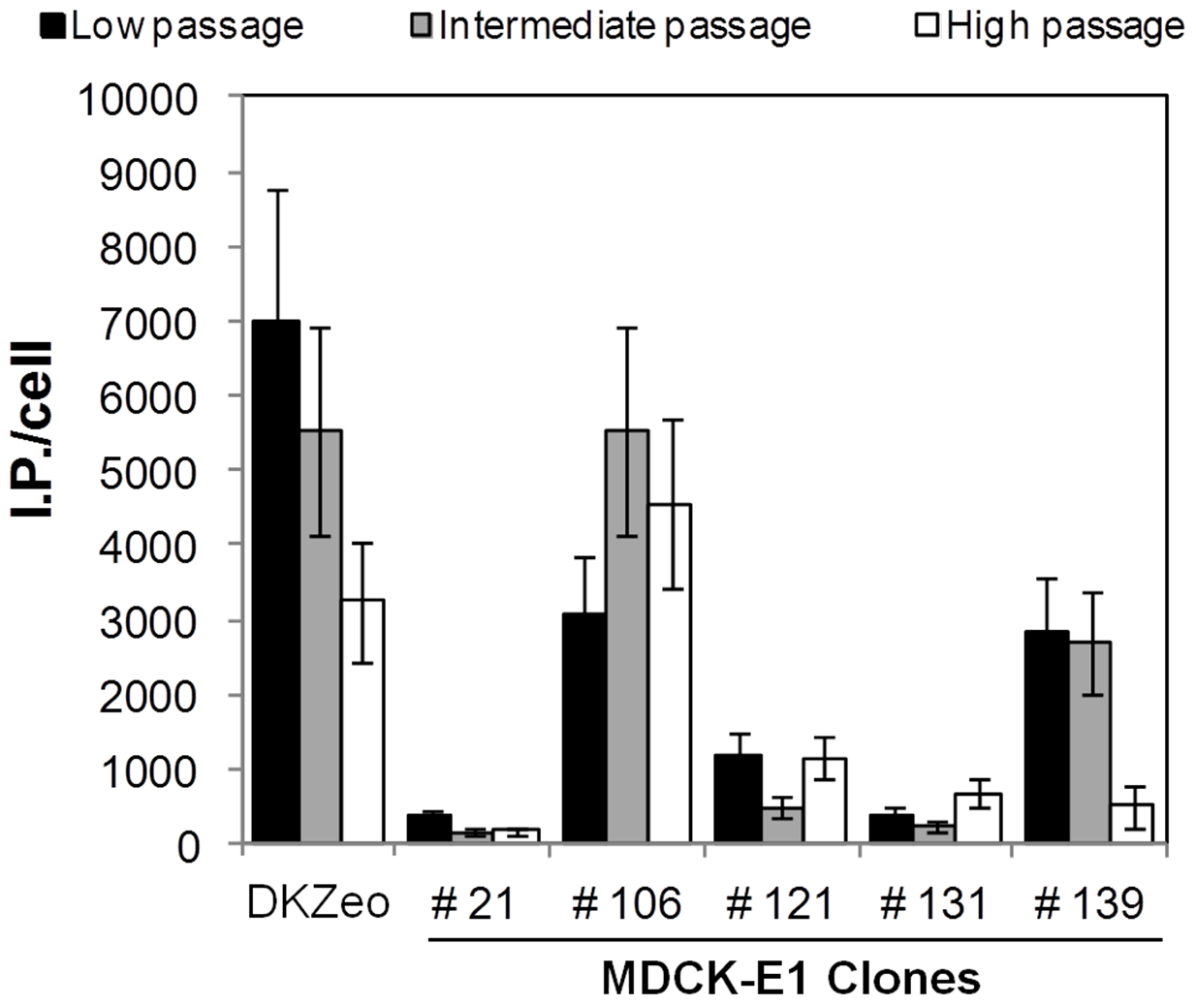
Cell specific viral titers obtained for MDCK-E1 cell clones and the control cell DKZeo at different cell culture passages, in order to assess its effect on the production of CAVGFP. Low passage corresponds to passage number 20, intermediate to passages between 30–40 and high passage to passages 45–55. Error bars represent a 25% inter-assay variability.

The long-term stability of E1 expression in MDCK-E1 clones was also assessed by analyzing cells without antibiotic selection pressure with increasing subcultures passages: low (passage 20), intermediate (passages 30–40) and high (passages 45–55) passages. Similar CAVGFP amplifications were obtained in the different cell passages of most MDCK-E1 clones tested. However, for MDCK-E1#139 clone, a decrease in the viral production was observed at high passages (**Figure 3**). Also, a 2-fold reduction in specific productivity of our reference cell line DKZeo [20] at high cell passage was observed. The highest cell culture passages tested for MDCK-E1#106 and DKZeo were 52 and 56, respectively.

**Figure 4:**
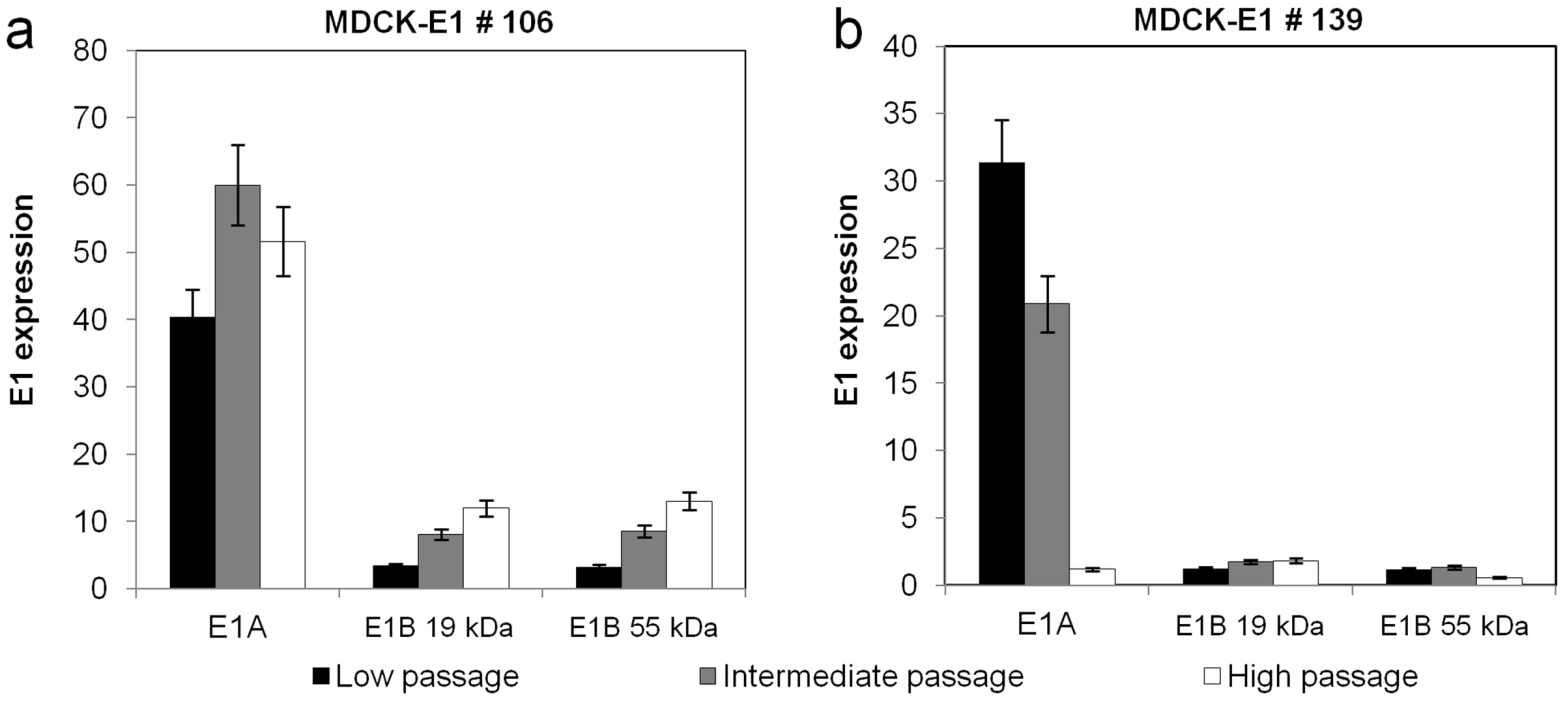
Levels of mRNA of E1A and E1B over increasing cell culture passages for MDCK-E1 clone #106 (a) and #139 (b). The gene expression was determined by Real Time reverse transcriptase PCR normalized to the housekeeping gene (GAPDH). Error bars represent a 10% variability error associated with the method.

E1 gene expression was determined in the three different ranges of passages for MDCK-E1#106 and MDCK-E1#139 (**Figure 4**). No significant variation in E1A expression with the increasing cell passages of MDCK-E1#106 was observed (**Figure 4a**). However, a gradual increase in E1B expression with increasing cell passages was observed for the same cell clone (**Figure 4a**). A clear reduction on E1A expression during subculture was observed for MDCK-E1#139, particularly at high passage, possibly explaining the drop in viral production for this clone (**Figure 4b**). The expression of E1B was maintained in MDCK-E1#139. A small decrease was observed in E1A expression of DKZeo cells at high passage (data not shown).

### Cre Expression in MDCK-E1 and HD CAV-2 Amplification

**Figure 5:**
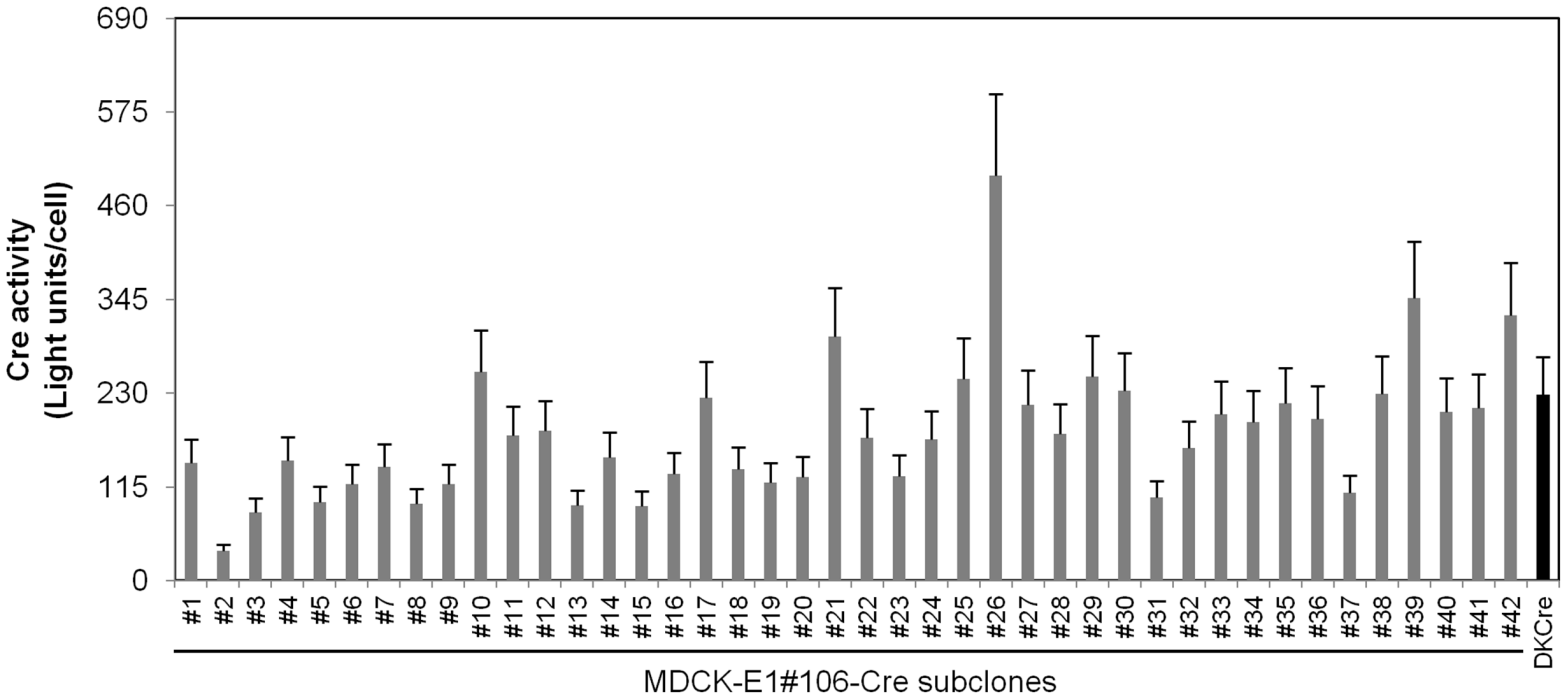
Cell specific Cre activity of MDCK-E1#106-Cre subclones and DKCre cells measured indirectly through a luciferase assay (see materials and methods). Error assumes 20% inter-assay variability.

To amplify helper-dependent (HD) CAV-2 vectors, and avoid helper-vector (HV) contamination, MDCK-E1 cells have to express Cre-recombinase. MDCK-E1#106, being the clone with the highest CAV-2 productivity (**Figures 2a** **and** **3**), was stably transfected with Cre recombinase gene and several clones were obtained. Cre activity was determined indirectly by infecting the subclones with AdMA19 [27], which when cleaved in the loxP site expresses luciferase (see materials and methods section), and compared with DKCre cells (**Figure 5**). In comparison to DKCre cells, 25% presented either equal or higher Cre activity. The subclones with higher Cre activity presented lower cell growth (data not shown).

To determine if high productivities are maintained after Cre expression, CAVGFP production was evaluated in five subclones with different Cre activities: Cre#2, Cre#10, Cre#19, Cre#16 and Cre#30. The results showed same range of productivities for all the subclones tested (**Table 2**).

**Table 2:** Amplification of CAVGFP and JBΔ5 in Cre-expressing clones and the corresponding parental cells.

Cells	Viral vectors				
	CAVGFP		JBΔ5		
	Amplification ratio	Productivity ratio	Amplification ratio	Fold decrease	Excision efficiency (%)
MDCK-E1#106	688±172	–	605±151	–	–
MDCK-E1#106-Cre#2	537±134	0.8	22±6	28	96
MDCK-E1#106-Cre#10	928±232	1.3	3±1	201	99
MDCK-E1#106-Cre#19	590±148	0.9	22±6	28	96
MDCK-E1#106-Cre#26	798±200	1.2	22±6	28	96
MDCK-E1#106-Cre#30	882±221	1.3	25±6	24	96
DKZeo	475±106	–	299±75	–	–
DKCre	604±151	1.3	10±3	30	97

The error in amplification ratio corresponds to 25% inter-assays variability. The productivity ratio corresponds to the ratio between CAVGFP amplification using Cre expressing and corresponding parental cells. Fold decrease in JBΔ5 production was calculated using the ratio between amplification value of parental cells (MDCK-E1#106 and DKZeo) and the corresponding Cre-expressing cells. Excision efficiency was calculated assuming that the difference in the amplification ratio of parental and corresponding Cre-expressing cells corresponds to unpackaged viral genomes.

In the current HDV production protocol it is fundamental to avoid HV contamination [13], [14], via Cre excision of the packaging signal of HV genome. However, a relative level of Cre activity is required to attain the effective excision levels. Thus, considering that subclones and the corresponding parental cells present same capability to produce viral vectors, we evaluated the excision efficiency of the packaging signal of JBΔ5 (CAV-2 HV), flanked by loxP, by analyzing its amplification in MDCK-E1-Cre subclones (**Table 2**). MDCK-E1#106-Cre subclones presented a reduced amplification capacity of ∼26-fold, which is in the range of that obtained with DKCre cells. MDCK-E1#106-Cre#10 was the exception, showing the highest fold-decrease among all MDCK-E1#106-Cre subclones. No evident relation was observed between the level of Cre activity and the amplification reduction occurring in cell subclones. Furthermore, all MDCK-E1-Cre subclones presented an estimated excision efficiency ≥96% (**Table 2**).

In order to establish the best conditions to produce HD CAV-2 vectors (maximum productivity and lowest HV contamination) before testing MDCK-E1-Cre subclones, production assays under different MOI ratios between HD CAVGFP and JBΔ5 (5 to 5, 5 to 1 and 5 to 0.5 I.P.) were performed using control DKCre cells. While under high MOI of JBΔ5 the specific productivity of HD CAVGFP was decreased to 3-fold with 60% of JBΔ5 I.P. in the final product, maximum production of HD CAVGFP and lowest contamination of JBΔ5 (ranging from 2–4%) were both obtained using the ratios 5 to 1 and 5 to 0.5.

To test the amplification of HD CAV-2 vectors in MDCK-E1-Cre cells, three subclones were co-infected with HD CAVGFP and JBΔ5 and evaluated regarding the production of HD CAVGFP and contamination level of JBΔ5. The selected MOI ratio was 5 to 1 to ensure at least one I.P. of JBΔ5 *per* cell. The results showed same productivity between the MDCK-E1-Cre subclones and DKCre cells, as well as a similar low contamination of HV (**Table 3**).

**Table 3:** Cell specific productivity of HD CAVGFP and corresponding contamination with helper JBΔ5.

Cells	HD CAVGFP		JBΔ5				
	I.P./cell	Amplification ratio	I.P./cell	Amplification ratio	Contamination (%)		
MDCK-E1#106-Cre#10	454±114	91±23	6±2	6±2		1.3	
MDCK-E1#106-Cre#26	469±117	94±24	8±2	8±2		1.6	
MDCK-E1#106-Cre#30	281±70	61±15	5±1	5±1		1.9	
DKCre	531±133	106±27	17±4	17±4		2.5	

Errors correspond to 25% inter-assay variability. The contamination levels of JBΔ5 correspond to the ratio between the I.P titers of JBΔ5 and HD CAVGFP.

### Physiological Implications of E1 and Cre on ΔE1 and HD CAV-2 Vectors Producer Cell Lines

Adenoviral E1B gene products are involved in the inhibition of apoptosis [8]–[10], thereby prolonging cell viability. On the other hand, Cre activity can induce growth inhibition and apoptosis [30]. To investigate the cellular response to the presence of E1 and Cre, death susceptibility under oxidative stress conditions was analyzed. MDCK-E1#106 and MDCK-E1#121 are shown as representatives of low and high E1B expression (**Figure 2c**), while MDCK-E1#106-Cre#10 and MDCK-E1#106-Cre#19 as representatives of high and low Cre activity (**Figure 5**), respectively.

**Figure 6:**
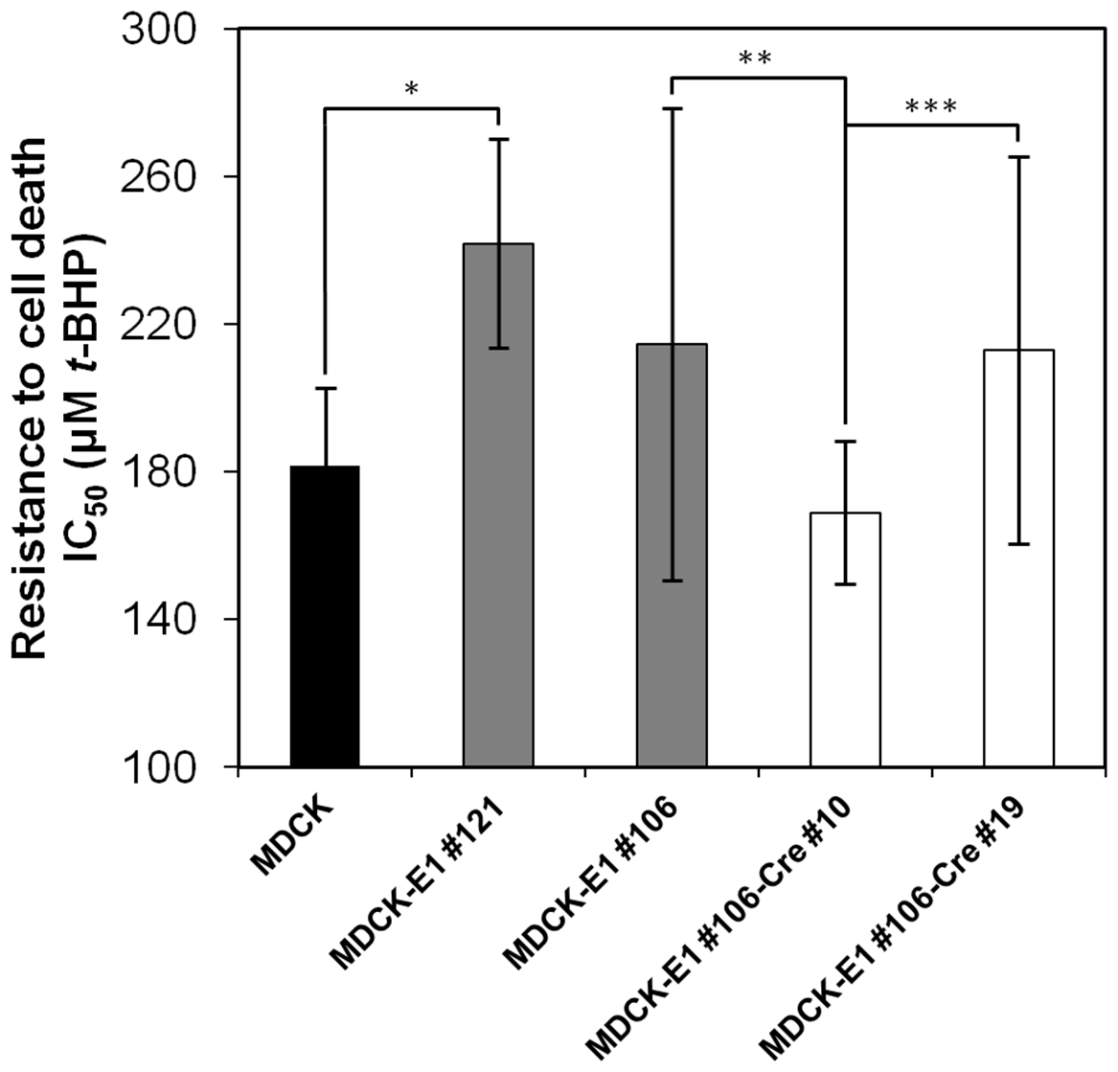
Half maximal inhibitory concentrations (IC50) of MDCK-E1 and MDCK-E1-Cre cell clones in response to increasing concentrations of *tert*-butyl hydroperoxide (*t*-BHP) (causing oxidative stress injury) in the culture medium. MDCK-E1#121 and MDCK-E1#106 represent high and low E1B expression, while MDCK-E1#106-Cre#10 and MDCK-E1#106-Cre#19 represent high and low Cre-activity, respectively. Error bars correspond to standard-deviation of quadruplicate assays. *p<4×10^−5^, **p<0.05, ***p<0.04, indicating the significance of a single factor Anova analysis.

Higher half maximal inhibitory concentration values (IC_50_) were obtained for MDCK-E1#121 clone (**Figure 6**). On the other hand, MDCK-E1#106-Cre#10, being the subclone with highest Cre activity, presented the highest susceptibility to oxidative stress. IC_50_ values of MDCK-E1#106-Cre#19, although higher than its counterpart (Cre#10) with higher Cre-activity, were not significantly different from either MDCK or MDCK-E1#106 cell clone. High levels of Cre in MDCK-E1#106 subclones resulted in an higher cell susceptibility to cell death that upon infection was translated in a decrease in cell density by approximately 10% after 48 hpi (data not shown). However, no significant impact in virus production was observed, as shown by the CAVGFP amplification yields that were maintained in MDCK-E1#106-Cre subclones (**Table 2**).

## Discussion

Most replication-defective AdV require E1 transcomplementing cell lines that provide E1A and E1B gene products essential for viral replication. The establishment of these producer cell lines is mainly focused in developing cells that allow high-yield production, while avoiding RCA generation [2]. To our knowledge, the impact of E1A and E1B expression levels on the production of AdV has never been investigated. To address this issue, and given the attractive features of these vectors to fundamental neurobiological questions and to develop potential treatment of neurodegenerative disorders [24], [25], [31], [32], we selected CAV-2 vectors production in MDCK-derived cell lines. Moreover, MDCK cells are already approved by the regulatory authorities for the manufacture of vaccines and thus representing a suitable cell substrate that might facilitate the regulatory approval for the production of clinical grade CAV-2 vectors [33]. Thus, the MDCK-derived cell lines developed herein, holding production yields similar to the ones obtained with the previously established DK cell lines, constitute a step forward for achieving that purpose.

This work confirms the similar functions of E1A and E1B gene products from canine and human adenoviruses, as extrapolated by sequence analogy. E1A encoded proteins from adenovirus play an essential role in viral production by acting as transcriptional activators of early viral genes [4], inducing cells to enter S-phase [34], [35] and allowing the use of cellular DNA replication machinery to support viral DNA replication [3], [36]. Therefore, it is not surprising that high production yields were attained under high levels of E1A transcripts (**Figures 2a,b**). When plotting E1A transcripts as a function of viral production from the different MDCK-E1 clones, a linear tendency was obtained (r^2^ = 0.99). MDCK-E1#121 was an exception: the higher expression of E1B seemed to compensate the intermediate level of E1A expression. MDCK-E1#121, showing the highest expression of E1B among all clones tested, also presented less susceptibility to oxidative stress injury (**Figure 6**) and higher cell concentration at virus harvesting time (**Figure 2d**). This indicates the involvement of E1B in cell ability to hold stressful conditions such as CAVGFP infection, which is supported by the functions dedicated to E1B gene products. E1B 19 kDa and E1B 55 kDa proteins from human adenovirus can independently inhibit the activation of apoptosis as a response to infection and foreign DNA replication [8], [37], [38]. E1B 55 kDa also inhibits cellular mRNA export and promotes viral mRNA export and translation during late infection [39]. Therefore, the high levels E1B expression had also an effect on viral production by extending cell viability. More interestingly, MDCK-E1#106 cell clone presented a concomitant increase on E1B expression with cell passages (**Figures 3** **and** **4a**). This gradual increase of expression can be due to an epigenetic mechanism started in a few cells, which conferred proliferating/anti-apoptotic selection advantage. Considering the main principle to choose cell lines (high production of AdV) and the direct relation between viral yields and E1A expression levels, the cell lines usually selected are likely to attain high levels of E1A. On the other hand, it is not guaranteed that these same cells hold appreciable E1B expression levels, due to its indirect role on viral yields. This has not a major impact on the production of E1-deleted AdV, however high expression of E1B should be pursued in scenarios where cell line has to further express toxic products, such as Cre recombinase during helper-dependent vectors (HDV) production.

Cre expression can result in a markedly reduced proliferation and genotoxic effects in cultured cells [30]. The aberrant activity on multiple pseudo loxP sites presented in mammalian genomes [40] induces DNA double-strand breaks (DSBs) or nicks that are converted to DSBs during DNA replication, leading to arrest of cells in G_2_/M phase because of intolerable DNA damage [30]. Indeed, MDCK-E1-Cre subclones with very high Cre activity presented reduced growth rates and we were unable to maintain some of them at long term in culture (data not shown). In addition to disturbed growth patterns, the subclones with high Cre activity become more susceptible to cell death (**Figure 6**), showing an average of 10% lower cell densities at virus harvesting time, in comparison with MDCK-E1 parental cells. The anti-apoptotic improvement conferred by E1B was decreased, and in this context, the impact of Cre should be further investigated under higher levels of E1B. Despite these observations, the MDCK-E1-Cre subclones used in this work attained reproducible growth rates and maximum cell concentrations similar to MDCK-E1 parental cells (data not shown). More important, similar CAV-2 vector production was observed between MDCK-E1-Cre and corresponding MDCK-E1 cells (**Table 2**). Although we hypothesized that the inhibitory effects of Cre could be extended to vector production, because adenoviruses inhibit key components of cellular DNA damage response [41], [42], compromising the repair of the Cre-induced DSBs, these findings showed no effect of Cre on infected cells productivity, probably due to the inhibition of DNA damage response taking place later in the lytic cyle [42].

The ideal situation to avoid cytotoxicity would be the minimum possible level of Cre that would ensure an efficient excision of the Ψ in the helper vector (HV) genome. Herein, the excision efficiency of the HV was assessed in subclones presenting different Cre activities (**Table 2**). Overall, all MDCK-E1#106-Cre cell subclones presented similar excision efficiency (above 96%), suggesting that above a minimal level of Cre, the excision of HV packaging signal is equal. However, in a situation where HV production is maximized, either by different viral construct or improved E1 expression of producer cell lines, more viral genomes would be replicated and the threshold of Cre for the same excision efficiency would probably increase. Nevertheless, under the conditions assayed herein, MDCK-E1-Cre cells are suitable producer cell lines, with similar excision efficiencies to the ones obtained for human HV [43]. Moreover, MDCK-E1-Cre clones attained appreciable HD CAV-2 titers; the HD CAVGFP amplification corresponded to a volumetric productivity of 1.5–4.0×10^8^ I.P./mL, which is similar to the one described for human HDV [44], [45]. The production of HD CAVGFP using 5 I.P./cell of this vector was compromised when co-infection was performed with high MOI of JBΔ5. In fact, the negative effect of higher MOI of HV on HDV yield, together with the generation of higher HV concentration, was already reported for human HDV by Dormond et al. [44]. Thus, lower MOI of JBΔ5 was used to test MDCK-E1-Cre clones. Under these conditions, lower amplification of HV was obtained, when comparing to what was observed in single infections with high MOI (**Tables 2** **and** **3**). This may be due to the use of a non-optimal MOI for HV replication or the presence of non-lethal mutations in HV packaging region [24], further reducing DNA packaging in co-infection scenarios [23], such as the production of HDV.

In this work, we show that the expression of E1 had an essential role in adenoviral vector production, using CAV-2 as a case study. While E1A levels had a direct effect on viral production, the expression of E1B conferred protection against cell death. The further activity of Cre, although impairing cell growth and physiology, had no effect on viral production and can be substantially reduced without compromising the excision of packaging signal from HV. These findings highlight the need to understand the influence of trans-complementing genes on the biology of host-cell and virus, providing important knowledge to choose and design superior cell lines and enhance adenovirus yields.
